# Consensus development of core competencies in intensive and critical care medicine training in China

**DOI:** 10.1186/s13054-016-1514-z

**Published:** 2016-10-16

**Authors:** Xiaoyun Hu, Xiuming Xi, Penglin Ma, Haibo Qiu, Kaijiang Yu, Yaoqing Tang, Chuanyun Qian, Qiang Fang, Yushan Wang, Xiangyou Yu, Yuan Xu, Bin Du, for the China Critical Care Clinical Trials Group (CCCCTG) and the Task Force of Core Competencies in Intensive and Critical Care Medicine Training in China

**Affiliations:** 1 Medical Intensive Care Unit, Peking Union Medicine Collage Hospital, 1 Shuai Fu Yuan, Beijing, 100730 China; 2 Department of Critical Care Medicine, Fuxing Hospital, Capital Medical University, Beijing, China; 3 Department of Emergency and Critical Care Medicine, People’s Liberation Army 309 Hospital, Beijing, China; 4 Department of Emergency and Critical Care Medicine, Zhongda Hospital, Southeast University, Nanjing, Jiangsu Province China; 5 Department of Critical Care Medicine, Haerbin Medical University Third Hospital, Haerbin, Heilongjiang Province China; 6 Department of Critical Care Medicine, RuiJin Hospital, Shanghai JiaoTong University School of Medicine, Shanghai, China; 7 Department of Emergency and Critical Care Medicine, Kunming Medical University 1st Hospital, Kunming, Yunnan Province China; 8 Department of Critical Care Medicine, Zhejiang University 1st Hospital, Hangzhou, Zhejiang Province China; 9 Department of Critical Care Medicine, Jilin University 1st Hospital, Changchun, Jilin Province China; 10 Department of Critical Care Medicine, Xinjiang Medical University 1st Hospital, Urumuqi, Xinjiang China; 11 Department of Critical Care Medicine, Beijing Tsinghua Changgung Hospital, Tsinghua University, Beijing, China

**Keywords:** Intensive care, Critical care, Training, Core competence, Delphi, Nominal group

## Abstract

**Background:**

The aim of this study is to develop consensus on core competencies required for postgraduate training in intensive care medicine.

**Methods:**

We used a combination of a modified Delphi method and a nominal group technique to create and modify the list of core competencies to ensure maximum consensus. Ideas were generated modified from Competency Based Training in Intensive Care Medicine in Europe collaboration (CoBaTrICE) core competencies. An online survey invited healthcare professionals, educators, and trainees to rate and comment on these competencies. The output from the online survey was edited and then reviewed by a nominal group of 13 intensive care professionals to identify each competence for importance. The resulting list was then recirculated in the nominal group for iterative rating.

**Results:**

The online survey yielded a list of 199 competencies for nominal group reviewing. After five rounds of rating, 129 competencies entered the final set defined as core competencies.

**Conclusions:**

We have generated a set of core competencies using a consensus technique which can serve as an indicator for training program development.

**Electronic supplementary material:**

The online version of this article (doi:10.1186/s13054-016-1514-z) contains supplementary material, which is available to authorized users.

## Background

Critical care medicine was recognized by the government as an independent specialty in China in 2008, almost three decades after its introduction in the 1980s [1]. Despite the lack of national census, it is a common belief that there has been great progress in critical care resources during the past 10 years; the provision of qualified intensivists through accredited training programs has therefore become a major challenge to meet increasing needs.

Postgraduate medical education in different fields of healthcare in China has been undergoing standardization for years, but standardized resident training in critical care is still under development. Up to 2010, there was no formal accredited critical care training program in China [1]. Moreover, there is no nationwide agreement upon evaluation and accreditation of critical care trainees, which makes it more difficult to attain the government objective of free movement of medical professionals as proposed by China’s healthcare reform plan [2].

Physician licensing has been slowly transforming from examination of knowledge to evaluation of competencies [3], which should develop during residency and fellowship training based on the Dreyfus model of knowledge development [4], and core competencies for graduates of fellowship programs in critical care have therefore been defined by multiple critical care societies in western countries [5–8]. In spring 2012, the Chinese College of Intensive and Critical Care Medicine (CCICCM) affiliated to the Chinese Medical Doctors’ Association (CMDA) called for a task force to define the minimum competencies of intensive and critical care training in China, using consensus techniques. The goal does not encompass developing a comprehensive curriculum inclusive of teaching techniques and assessment methods, but to allow individual training centers to harmonize their training program or curriculum focused on producing intensive care specialists with common core skills. The present article describes the process and outcome related to this mandate, and we wish to set an example for such efforts in low and middle-income countries.

## Methods

We used a combination of a modified Delphi method and a nominal group (NG) to generate and rate the importance of core competencies for critical care training [9, 10]; this approach has been used successfully by others to develop competency-based critical care training [5–8]. The Delphi technique, originally developed in the 1950s at The RAND Corporation, is designed to gather input from expert contributors using an iterative process with feedback of individual and group ratings for each item until full consensus is achieved. The modified Delphi method consists of beginning the process with a set of carefully selected items, which are drawn from various sources including related competency profiles, synthesized reviews of the literature, and interviews with selected content experts. The NG technique uses a small number of people with a facilitator to encourage sharing and discussion of reasons for the choices made by each group member, thus permitting consideration of concepts in depth [10].

There were three phases in the process. Phase 1 used a web-based survey to generate ideas for competencies categorized as certification, knowledge, procedures, and skills. Phase 2 used a NG to rate the edited list of competencies iteratively. Phase 3 involved recirculation of the results for further comment.

### Phase 1: generation and structuring of competencies

We developed the list of potential core competencies based on those proposed by the Competency Based Training in Intensive Care Medicine in Europe collaboration (CoBaTrICE) [6], the clinical roles of intensivists defined by Society of Critical Care Medicine (SCCM) [11], and the guidelines for critical care medicine training and continuing medical education developed by the American College of Critical Care Medicine (ACCM) [12]. Modifications of the original items were made, if necessary, to improve clarity or provide greater detail. For example, we extended the original item “3.1 Manages the care of the critically ill patients with specific acute medical conditions” in the CoBaTrICE competency list to a group of specific diseases and/or clinical syndromes.

The online questionnaire consisted of four parts: characteristics of respondents; a list of specific credentials available in China, such as basic life support (BLS); the list of potential core competencies categorized as knowledge, skills, and behaviors/attitudes, with the respondents asked to check “YES” or “NO” for each item; and an open question invited the respondents to define “what competencies … are important for intensivists, apart from those listed above”. The final questionnaire was posted on the official website of the Chinese Society of Critical Care Medicine (CSCCM) (http://www.csccm.org.cn) on November 27, 2012, and was open to feedback and comments until January 3, 2013. An invitation letter with the objective of the study and the website link to the questionnaire was sent by email according to the CSCCM membership database, supplemented by a regional and personal snowball method.

Results of the web-based survey were integrated to generate a formal list including all those items receiving at least one “YES” response. In addition, explicit responses to the open question were rephrased based on consensus among the investigators and included in the list.

### Phase 2: NG rating

The planned NG comprised 10 intensivists, two fellow trainees in critical care, one respiratory therapist, and one registered critical care nurse, which mimicked the composition of online survey respondents. The three national critical care societies – the CCICCM, the CSCCM affiliated to the Chinese Association of Pathophysiology, and the Chinese Society of Intensive Care Medicine (CSICM) affiliated to the Chinese Medical Association (CMA) – were asked to nominate several senior intensivists from each party. These nominated intensivists were supposed to be experts in critical care trainings, such as directors of fellowship programs in critical care in university hospitals. The rest of the NG members were randomly selected from online survey respondents, while intensivists practicing for less than 3 years in ICU were identified as trainees. The list of planned NG members was reviewed and approved with consensus by the leadership of these three national societies, with the consideration of geographic distribution. However, one intensivist did not respond to the invitation, and therefore there were 13 members of the final NG (Additional file 1: Table S1), all from university hospitals. All NG members were working remotely and remained blinded to the composition of the NG throughout the study. One author (XH), an intensivist familiar with consensus techniques, worked as the study coordinator, in charge of communication with participants individually by email or telephone to ensure data accuracy without participating in the rating process.

An electronic questionnaire of suggested competencies was sent to all NG members by email. All of the competencies shared one stem: “At the end of training, the trainees should be able to …”. For each item, the proportion of respondents checking “YES” in the online survey during Phase 1 was also provided. A descriptive document was attached to the questionnaire, including a brief introduction of the project, instruction for rating, and contact information of the task force. We also reminded NG members to consider the well-recognized variation of critical care resources in different hospitals and geographic regions across China. The NG members were asked to rate individual competencies using a 5-point Likert scale (ranging from 1 = strongly disagree to 5 = strongly agree), and to return the questionnaire within 1 week.

All returned questionnaires were manually reviewed for any missed items or inconsistencies, and then the coordinator would contact the respondent by email or telephone for clarification if necessary. All responses to individual items were analyzed using predefined consensus and cutoff criteria. Items achieving full consensus (100 % of respondents agreed or strongly agreed) were removed from further iteration and entered into the final set. Items were rejected directly if none of the respondents chose agree or strongly agree. Other items went through to the next round. Information about personal and group ratings for each item during the previous rounds were provided for reference. Each round was finished within 8 weeks.

After three rounds, one-third of items were still without full agreement. Upon discussion with all NG members, we thought that further agreement might be achieved by clarifying the level of expertise at which the competency should be held. Therefore, we performed another two rounds of rating using items without full agreement. For each item, we asked the NG members to respond to the question “at what level of expertise do you think this competency is required”, using the CoBaTrICE approach to describe the level of expertise in the competence statements [6], which was expressed on a modified 5-point Likert scale (1 = no need to understand; 2 = has the knowledge or describes; 3 = performs, manages or demonstrates … under supervision; 4 = performs, manages, or demonstrates … independently; 5 = teaches or supervises others in the performance, management, or demonstration). The same consensus and cutoff criteria were used in last two rounds; that is, any items achieving full consensus (Likert scale scores of 4 and 5) would enter the final set.

### Phase 3: recirculation for comment and iterative review

The output from the NG ratings was posted on the official website of the CSCCM (http://www.csccm.org.cn) for 1 month, with a shared link posted on both the CCICCM and the CSICM websites at the same time [5, 6]. Because the three national critical care societies had a 90 % overlap in membership, and the CSCCM had most intact corresponding information, all members of the CSCCM membership database were contacted by email and invited to provide free-text commentary. Comments might include any disagreement with the NG ratings, or any further suggestions that rejected items should be incorporated into the final set.

### Statistical analysis

Demographic data and rating scores were presented as the mean and 95 % CI. The percentage of agreement was defined as the percentage of respondents rating agree or strongly agree for each item. We used kappa and Kendall *W* statistics to compare inter-rater consistency of each round.

## Results

### Phase 1: generation and structuring of competencies

During the 2-month period, 398 participants completed the online survey. Most of the respondents (91 %) were physicians, with 81 % as intensivists and 10 % as other specialists such as emergency physicians, pulmonologists, anesthesiologists, and surgeons. The remaining respondents included medical students (5 %), nurses (2 %), and respiratory therapists (2 %). Among the intensivists, 39 % had been practicing critical care medicine for more than 10 years, while 16 % spent no more than 3 years working in the ICU.

All items in the original online questionnaire received more than one rating, in addition to 65 suggestions from the open question, all of which but one (i.e. molecular adsorbent recirculating system (MARS)) were already included in the existing items. After meticulous examination of all suggestions and minor modifications of terminology, a total of 199 statements were generated for NG rating. The list was composed of three suggested specific credentials required for each trainee to achieve at the end of training and 196 competency stems. All competency stems were expressed as a combination of key words of competency and a descriptive phrase indicating on which domain the competency should be evaluated (Table 1). The level of expertise each item required was not included in the statement.

**Table 1:** Competence statement construction

Domain			Key words
Knowledge	Skills	Attitudes/behaviors	
Assesses	Manages	Explains	Description of competency: e.g., … initial management of the trauma patient e.g., … bag-mask ventilation
Describes	Provides	Appraises	
Interprets	Integrates	Complies	
Orders	Obtains	Demonstrates	
Recognizes	Performs	Ensures	
Identifies	Administers	Involves	
	Maintains	Promotes	
	Communicates	Respects	
	Prepares	Takes responsibility	
	Applies	Supports	
	Organizes	Participates	
	Formulates		

### Phase 2: NG rating

**Fig. 1:**
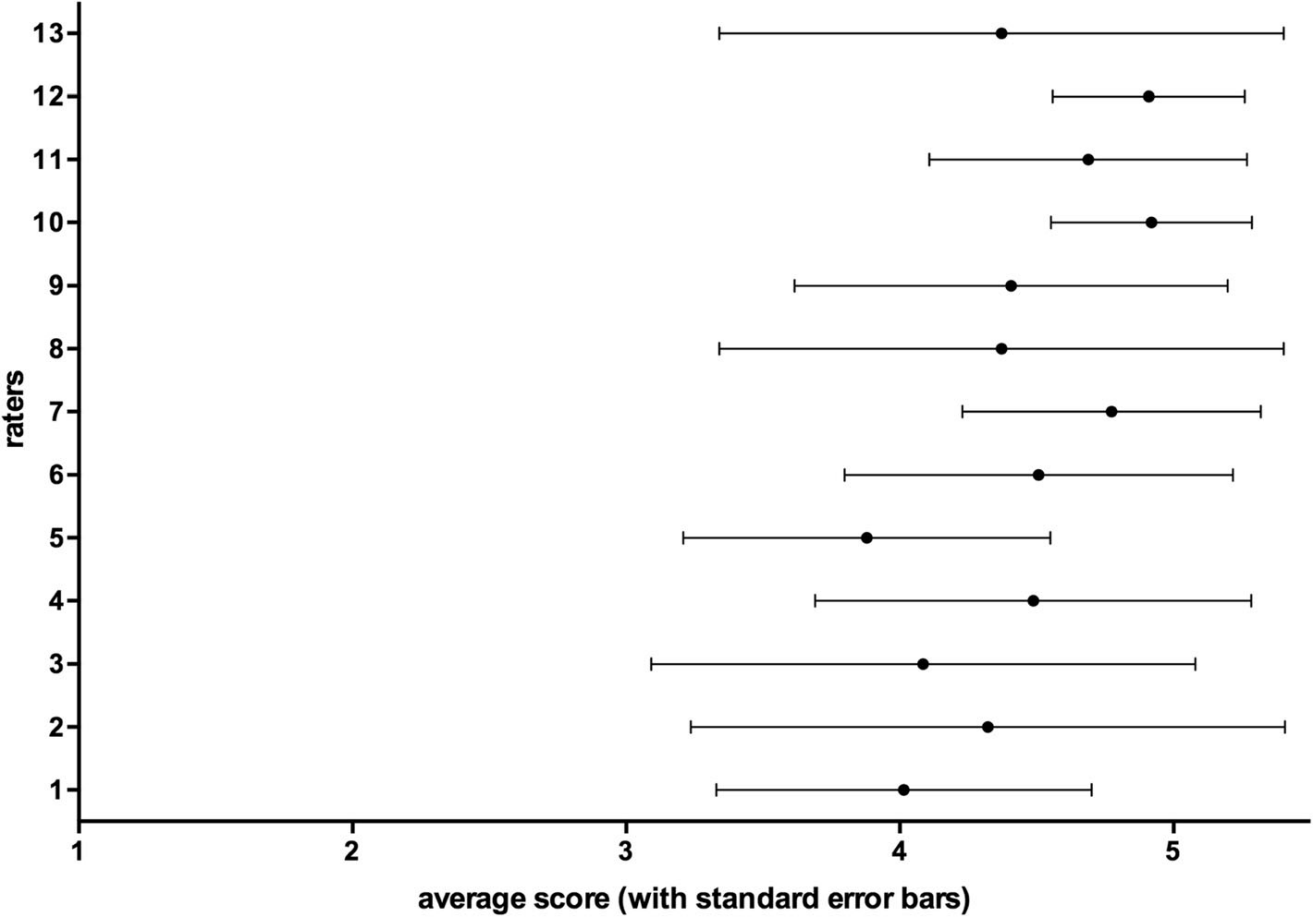
Average (*dot*) and standard error (*error bars*) for rating points in the first-round rating for each rater in the NG

**Fig. 2:**
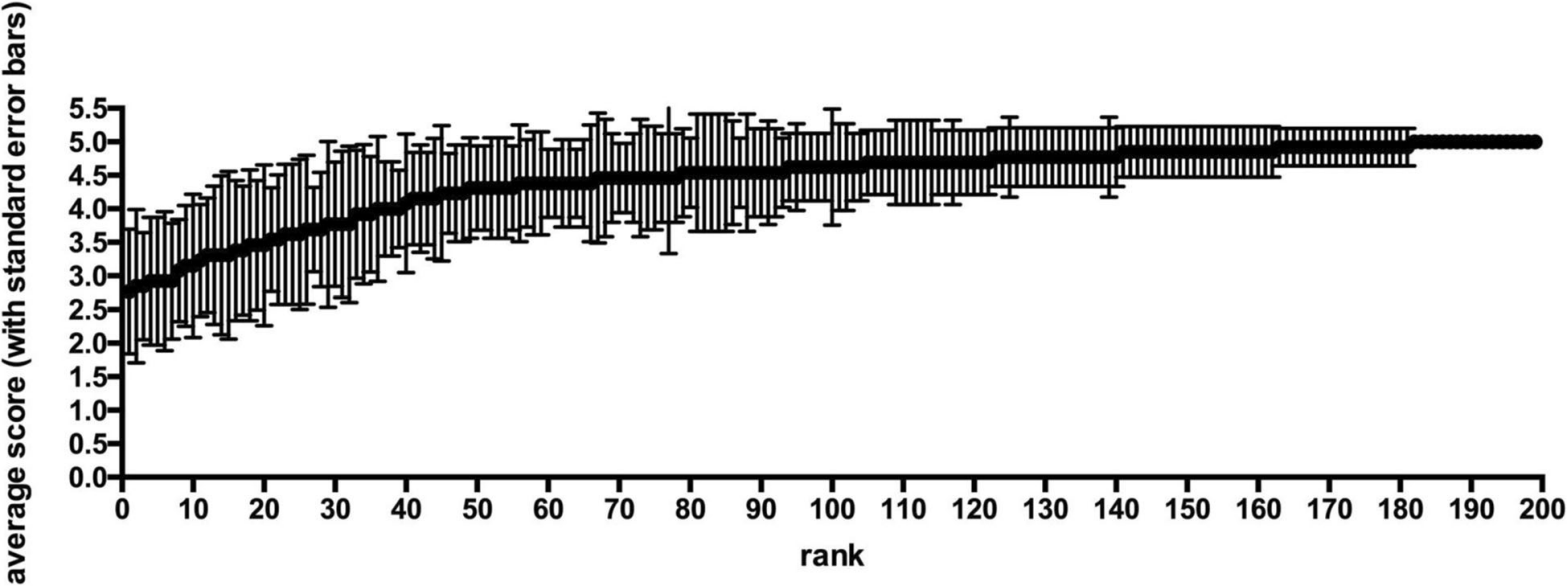
Mean Likert score (*line*) and standard deviation (*error bars*) for each of the competency statements resulting from the first-round NG rating

During the first round, the mean rating scores of individual NG members for all 199 items ranged from 3.88 to 4.92 (Fig. 1), yielding 101 items with full agreement (Fig. 2). During the second and third rounds, 20 and 8 additional items achieved full agreement, respectively (Table 1). Only one item (i.e., MARS) was rejected after the third round.

Overall agreement among the NG members in item rating was poor, despite a mild improvement from round one (κ = 0.36, Kendall *W* = 0.52) to round three (κ = 0.43, Kendall *W* = 0.58). Although there was significant variation in the mean rating score, no correlation was found between the rating score and the academic status or clinical experience of the respondent, except that ratings by the respiratory therapist were significantly lower than those by the senior intensivists in most rounds. However, there was a trend for greater agreement between NG members for the more highly rated competencies, with less agreement for those competencies that attracted lower levels of support (Fig. 2).

We decided to proceed to two more rounds with adjustments of the scoring system to focus on the level of expertise required for each competency. Although statistics showed improved agreement (κ = 0.65, Kendall *W* = 0.97 after the fifth round), the last two rounds did not yield any item with full agreement.

### Phase 3: recirculation

During the 1-month consultation period, although there were more than 2000 views of the webpage, only 23 detailed responses were received, all from NG members. One response raised concerns about too many competencies in the list, which might be difficult for regions with limited resources to achieve. The other responses proposed upgrade importance or level of expertise of several competencies. No additional topics were proposed.

### Development of the final set

All 129 items with full agreement were put into the final set (Table 2), after exclusion of 70 items (Additional file 1: Table S2). The items were grouped into 11 themes, one of which was credentials required at the end of training; others were core competencies required as critical care specialists, which means the trainees should be able to practice independently at the end of training. Minor modification to the terminology of several competencies was made to clarify the role of the intensivists in complicated conditions. For example, we added “due to infection or foreign body” to the original item “Assesses and manages upper airway obstruction”. Themes considered important by the NG during iterative rating and recirculation were included by modifying the expression. We added “including informed consent and end-of-life care” to the item “Involves patients (or their surrogates if applicable) in decisions about care and treatment”. The reason for the modification is that, while all NG members agreed that end-of-life care is important, some were concerned that trainees would be unable to achieve some of the items in this theme due to limited training resources compounded by cultural issues.

**Table 2:** Final set of core competencies required for critical care specialists and the supporting rate for each item

Theme	Competency statement	Online survey (%)	Agreement (%)^a^		
			Round one	Round two	Round three
1. Resuscitation and initial management of acutely ill patient	1.1 Assesses and provides initial management of the trauma patient	95.0	100		
	1.2 Manages cardiopulmonary resuscitation	99.0	100		
	1.3 Manages postresuscitation brain protection	99.0	100		
	1.4 Provides advanced life support for postresuscitation patient	99.7	100		
2. Diagnosis, assessment, investigation, monitoring, and data interpretation	2.1 Obtains medical history and performs accurate physical examination	99.0	100		
	2.2 Orders timely and appropriate laboratory investigations	99.2	100		
	2.3 Orders timely and appropriate image investigations	98.5	100		
	2.4 Integrates clinical findings with laboratory investigations to form a differential diagnosis	97.5	92	100	
	2.5 Describes and assesses patient with severity-of-illness score: APACHE, SAPS	96.2	100		
	2.6 Describes and assesses patient with multiorgan dysfunction score: SOFA, MODS	90.7	100		
	2.7 Describes monitoring and interpretation of respiratory mechanics	97.2	100		
	2.8 Interprets arterial blood gas analysis	95.5	92	100	
	2.9 Performs electrocardiography and interprets the results	88.4	100		
	2.10 Interprets chest radiographs	89.7	100		
	2.11 Interprets CT image	75.1	92	100	
3. Disease management	3.1 Describes implications of chronic and comorbid disease in the acutely ill patient	96.7	85	100	
	3.2 Recognizes and manages different types of shock	99.7	100		
	3.3 Assesses and manages life-threatening arrhythmia	99.7	100		
	3.4 Recognized and manages left ventricular failure and/or acute pulmonary edema	99.7	100		
	3.5 Recognizes and manages right heart failure	97.2	100		
	3.6 Assesses and manages myocardial infarction and acute coronary syndrome	97.5	100		
	3.7 Recognizes and manages rupture of aneurysm (bleeding and cardiac tamponade)	91.5	100		
	3.8 Recognizes and manages hypertension crisis	98.7	100		
	3.9 Describes physiological changes of cardiovascular system under acute condition	98.2	100		
	3.10 Assesses and manages acute and chronic respiratory failure	99.5	100		
	3.11 Assesses and manages acute exacerbation of chronic obstructive pulmonary disease	98.7	100		
	3.12 Assesses and manages status asthmaticus	99.5	100		
	3.13 Assesses and manages smoke inhalation, airway burns	96.0	100		
	3.14 Assesses and manages upper airway obstruction (due to infection or foreign body)	97.0	100		
	3.15 Recognizes (diagnosis and grading) and manages ARDS	99.7	100		
	3.16 Manages life-threatening hemoptysis	97.2	100		
	3.17 Describes effects of positioning on respiratory physiology	95.5	92	100	
	3.18 Recognizes (diagnosis and grading) and manages acute kidney injury	99.0	100		
	3.19 Manages critically ill patients with chronic renal failure	97.7	100		
	3.20 Manages patients with coma	100.0	100		
	3.21 Assesses and manages patients with drug overdose and intoxication	96.2	92	100	
	3.22 Assesses and manages cerebral vascular accident	97.5	92	100	
	3.23 Manages status epilepticus	97.7	92	100	
	3.24 Recognizes and manages intracranial infection	98.0	92	100	
	3.25 Assesses and manages patient with increased intracranial pressure	98.5	92	100	
	3.26 Assesses and manages spine injury	91.5	85	92	100
	3.27 Recognizes and manages adrenal crisis	95.2	100		
	3.28 Recognizes and manages diabetes insipidus	92.5	100		
	3.29 Recognizes and manages diabetic ketoacidosis	99.5	100		
	3.30 Recognizes and manages sepsis, severe sepsis, and septic shock	100.0	100		
	3.31 Assesses and manages multiorgan dysfunction syndrome	99.7	100		
	3.32 Assesses and manages severe community acquired infection (e.g., severe community-acquired pneumonia)	98.7	100		
	3.33 Recognizes and manages nosocomial infection	99.0	100		
	3.34 Assesses and manages fever in critically ill patient	96.0	100		
	3.35 Describes antimicrobial resistance	99.7	100		
	3.36 Recognizes intra-abdominal infection and gastrointestinal leakage	96.5	100		
	3.37 Manages coagulopathy	99.0	100		
	3.38 Manages hemolytic disorders	89.7	92	92	100
	3.39 Assesses and manages thromboembolic disease (including pulmonary embolism)	99.2	100		
	3.40 Manages disseminated intravascular coagulation	98.5	92	100	
	3.41 Manages traumatic coagulopathy	95.7	92	92	100
	3.42 Assesses and manages gastrointestinal bleeding	98.7	100		
	3.43 Assesses and manages patient with liver failure	97.7	100		
	3.44 Assesses and manages pancreatitis	99.2	100		
	3.45 Assesses and manages abdominal compartment syndrome	97.2	100		
	3.46 Assesses and manages acute illness in pregnancy	93.0	100		
4. Therapeutic interventions/organ system support in single or multiple organ failure	4.1 Assesses and manages fluid and electrolyte disorders	99.7	100		
	4.2 Assesses and manages acid–base disorders	98.7	100		
	4.3 Describes and provides parenteral nutrition support	99.0	100		
	4.4 Describes and provides enteral nutrition support	98.7	100		
	4.5 Provides nutrition support for patient with severe acute pancreatitis	97.0	92	100	
	4.6 Provides nutrition support for patient with renal failure	97.0	92	92	100
	4.7 Provides nutrition support for patient with liver failure	96.7	92	100	
	4.8 Provides nutrition support for patient with sepsis and septic shock	98.5	92	100	
	4.9 Provides nutrition support for postgastrointestinal surgery patient	94.7	92	92	100
	4.10 Assesses and manages pain in critically ill patients	98.5	100		
	4.11 Describes principle and assessment of sedation	99.5	100		
	4.12 Provides assessment, prevention, and treatment of delirium	96.7	100		
	4.13 Describes indication and choice of neuromuscular blockade	92.5	92	92	100
	4.14 Manages fluid therapy	99.7	100		
	4.15 Manages vasoactive/inotropic medication therapy	100.0	100		
	4.16 Describes principles of drug dose adjustment in renal failure	98.7	100		
	4.17 Describes principles of continuous renal replacement therapy	99.0	100		
	4.18 Explains and appraises management of severe sepsis and septic shock	99.7	100		
	4.19 Describes principle of antimicrobial agent selection and dosing in critically ill patients	100.0	100		
	4.20 Describes principle of anticoagulation; anti-fibrinolytic therapy	98.0	100		
	4.21 Describes principle of blood component transfusion	99.2	100		
	4.22 Describes stress ulcer prophylaxis	99.2	92	100	
5. Practical procedures	5.1 Performs bedside ultrasound to localize pleural effusion and ascites	73.6	100		
	5.2 Maintains an open airway in the nonintubated patient	98.5	100		
	5.3 Performs bag-mask ventilation	98.5	100		
	5.4 Performs tracheal intubation	97.7	100		
	5.5 Performs tracheal aspiration	95.5	100		
	5.6 Manages pneumothorax	84.2	100		
	5.7 Administers oxygen therapy	98.2	100		
	5.8 Manages noninvasive and invasive mechanical ventilation: indication, rational, complication, and weaning	98.0	100		
	5.9 Explains and performs recruitment maneuver: principle and practice	95.2	100		
	5.10 Performs arterial puncture and cannulation	96.5	100		
	5.11 Performs central venous catheter insertion	97.7	100		
	5.12 Performs and interprets cardiac output and hemodynamic monitor	93.7	100		
	5.13 Performs cardioversion and defibrillation	97.7	100		
	5.14 Performs lumber puncture	87.9	100		
	5.15 Performs nasogastric tube placement	83.7	100		
	5.16 Performs abdominal paracentesis	96.2	100		
	5.17 Performs and interprets intra-abdominal pressure monitor	86.9	100		
	5.18 Manages continuous renal replacement therapy	91.5	100		
	5.19 Performs urinary catheterization	82.9	92	100	
6. Perioperative care	6.1 Performs preoperative cardiopulmonary evaluation of high-risk patient	92.7	92	100	
	6.2 Manages postoperative assessment and care of high-risk surgical patient	98.2	100		
	6.3 Manages the preoperative and postoperative care of the trauma patient	94.2	77	77	100
7. Comfort, recovery, and end-of-life care	7.1 Describes and applies practice to minimizes the physical and psychosocial consequences of critical illness for patients and families	98.5	100		
	7.2 Manages the safe and timely discharge of patients from the ICU	98.5	100		
	7.3 Communicates the continuing care requirements of patients at ICU discharge to healthcare professionals, patients, and relatives	96.2	100		
8. Transport	8.1 Assesses patient before transport	99.0	100		
	8.2 Prepares equipment for transport	98.0	100		
	8.3 Performs intrahospital transport	97.5	100		
9. Patient safety and system management	9.1 Complies with infection control measures	99.0	100		
	9.2 Identifies environmental hazards and promotes safety for patients and staff	97.2	92	100	
	9.3 Organizes a case conference	98.0	100		
	9.4 Critically appraises and applies guidelines, protocols, and care bundles	97.7	92	100	
10. Professionalism	10.1 Formulates clinical decisions with respect for ethical and legal principles	95.0	100		
	10.2 Involves patients (or their surrogates if applicable) in decisions about care and treatment (including informed consent and end-of-life care)	91.0	92	85	100
	10.3 Demonstrates respect of cultural and religious beliefs and an awareness of their impact on decision-making	94.0	92	100	
	10.4 Promotes effective team working	97.2	100		
	10.5 Communicates effectively with patients and relatives	97.7	100		
	10.6 Communicates effectively with members of the healthcare team	97.7	100		
	10.7 Maintains accurate medical records and documentation	98.0	100		
	10.8 Respects privacy, dignity, confidentiality, and legal constraints on the use of patient data	97.2	100		
	10.9 Takes responsibility for safe patient care	97.7	100		
	10.10 Ensures continuity of care through effective handover of clinical information	97.7	100		
	10.11 Seeks learning opportunities and integrates new knowledge into clinical practice	97.7	100		
	10.12 Describes and explains the managerial and administrative responsibilities of the ICM specialist	97.2	100		
11. Certification	11.1 Basic life support	99.0	100		
	11.2 Advanced cardiac life support	98.2	100		

*APACHE* Acute Physiological and Chronic Health Evaluation, *SAPS* Simplified Acute Physiological Score, *SOFA* Sequential Organ Failure Assessment, *MODS* Multiple Organ Dysfunction Score, *CT* computerized tomography, *ARDS* acute respiratory distress syndrome, *ICU* intensive care unit

^a^Percentage of respondents rating agree or strongly agree

We also compared our final set with those core competencies generated by the CoBaTrICE. Our list included more items in the domains such as disease management and therapeutic intervention/organ system support, but fewer items in domains involving practice procedures, perioperative care, end-of life care, and system management (detail shown in Additional file 1: Table S3). More items in ‘disease management’ were included in the final set than items in ‘practical procedures’ (Table 3).

**Table 3:** Proposed competencies categorized according to theme

Theme	Number of competencies entered for NG rating	Number of competencies rejected	Number of competencies accepted	Number of competencies in CoBaTrICE list	Number of competencies in both lists
1. Resuscitation and initial management of acutely ill patient	7	3	4	6	3
2. Diagnosis, assessment, investigation, monitoring, and data interpretation	14	3	11	12	8
3. Disease management	62	16	46	10	10
4. Therapeutic interventions/organ system support in single or multiple organ failure	26	4	22	13	13
5. Practical procedures	47	28	19	26	13
6. Perioperative care	7	4	3	6	3
7. Comfort, recovery, and end-of-life care	8	5	3	7	3
8. Transport	4	1	3	1	1
9. Patient safety and system management	5	1	4	8	4
10. Professionalism	16	4	12	15	11
11. Certification	3	1	2	0	0
Total	199	70	129	104	69

*CoBaTrICE* Competency Based Training in Intensive Care Medicine in Europe collaboration, *NG* nominal group

## Discussion

Leadership in intensive and critical care medicine in mainland China convened a broad array of clinical experts, credentialing and certifying bodies, and all national critical care societies. We used consensus techniques to develop a set of core competencies for intensive and critical care medicine training, which have been approved by representatives of all national critical care organizations. This is the first time that such a consensus in ICM has been developed in China.

The Delphi technique has been used in the field of healthcare education and training since the 1990s. One of the major advantages that make this method so popular is the ability to allow participants from different geographic regions to share their opinion with each other, and revise them by iteration. In addition, our study used the NG methodology, which has been validated to represent the views of the wider critical care community in developing national research priorities [9, 13]. Moreover, we made efforts to minimize bias, because all NG members in our study remained blinded to the composition of the NG as well as the individual response of any other NG members. Although a methodology involving face-to-face meetings may have led to greater agreement, while working remotely might compromise the level of agreement and may also be more time consuming, this approach may minimize the “tyranny of majority” during the rating process. In the meantime, keeping raters blind to each other during iteration has been well documented in several similar situations [14, 15].

It is noteworthy that there are significant differences in the core competencies developed in this study and those by the CoBaTrICE (Table 3, and Additional file 1: Table S3), highlighting the different insights of the Chinese intensive care community from those of Europe and North America [5–8]. Possible explanations for such differences may vary depending on different themes and merit further investigation. In consideration of heterogeneity of diseases and uneven distribution of healthcare resources all over the nation, we tried to determine a detailed requirement of basic knowledge and skills under certain acute conditions instead of a package of proposal that “manages the care of the critically ill patient with specific acute medical conditions” [6]. A list of 62 specific diseases and conditions were included in the initial questionnaire and yielded 46 items in the final set. Despite the differences in the level of focuses (disease level vs organ system level), our list and the CoBaTrICE syllabus covered almost the same spectrum of acute and critical conditions in the domains of disease management and therapeutic intervention/organ system support. The considerably shorter list of practice procedures might reflect the limitation of training resources in our country. Competencies related to catastrophe management were not included, because some NG members believed that emergency physicians and nurses should be more involved. Despite a similar enthusiasm for professionalism to the CoBaTrICE study [6], an unexpected result is that issues related to end-of-life care such as palliative care and brainstem death testing were rated less important than in Europe and North America. Data from rounds four and five showed that some NG members believed these items should be performed under proper supervision until the end of training. One possible reason is that end-of-life care in China is far underdeveloped, which limited the training sources for these parts [16, 17].

The core competencies generated from our study should be considered minimum requirements of intensive and critical care training which should be used to define a qualified intensivist, whereas those submitted to the NG for initial review but excluded during iterative rating might serve as optional competencies. We used a standardized descriptive term in each statement to clarify at which level the competence should be evaluated. Knowledge and skills can be evaluated by multiple-choice questions (MCQs) and objective structured clinical examination (OSCE) with high reliability [18], while portfolios and faculty rating are more used in attitude and behavior assessment [19]. We understand that some contents need to be revised, expanded, or added, highlighting the need for continuous reviewing and updating in the future. Our ultimate goal is to produce a standardized curriculum and evaluation system for intensive and critical care training, a time-consuming process requiring a more detailed guideline. However, the final set at the current stage should still be considered a starting point—that is, the fundamental standards that may guide future education goals and professional development in intensive and critical care specialty across the whole nation—and should allow curriculum managers to use these competencies as building blocks to develop a curriculum responsive to any special local training needs.

Our study has a few limitations. First, due to limited human resources and time boundaries, membership of the NG was quite small. Moreover, all members were selected from university hospitals, and should not be considered representative of the whole nation. However, almost all of the training bases for resident standardized training programs, which were approved by the Ministry of Health, were university hospitals. We understand that in the future many of the trainees will end up working in smaller or community hospitals, where competency requirements might be different from those in university hospitals. We thus kept reminding NG members to consider resource diversity of the national medical system through each round of rating. Our results show that 19 out of 47 (40.4 %) items in “practical procedures” were selected, whereas 46 of 62 (74.1 %) items in “disease management” were chosen. This may indicate that most competencies requiring advanced training resources were excluded from the final set.

Another limitation is that we did not invite patients or patient families to participate in our survey, which might lead to less attention to the opinions of “consumers”. However, our questionnaire was based on core competencies in the literature, including those generated by the CoBaTrICE [6]. The CoBaTrICE coordinators used a separate survey questionnaire including items about communication and interpersonal skills, and decision-making in addition to medical knowledge and skills, to seek for views from ICU patients and their relatives. Responses from this survey were also integrated with those from medical professionals during their NG rating [6]. However, the NG rating and subsequent iteration process involved only healthcare stakeholders. Therefore, our result of less attention to several ethical issues such as palliative care cannot be explained by an absence of consumers’ opinion during the whole process.

One last limitation is that during the recirculation phase (Phase 3) we did not receive a response from anyone other than NG members, which was unexpected. This may cast doubt on the usefulness of this phase.

## Conclusion

Using a consensus technique, we defined a list of core competencies required for intensive and critical care training in China. Further development of detailed syllabus and guidelines for training programs should target these goals.

## Additional file

Additional file 1: is **Table S1.** Presenting detailed information for members of the NG, **Table S2.** Presenting items not identified as core competencies, and **Table S3.** Presenting a comparison of core competencies generated by the CCCCTG and CoBaTrICE. (DOCX 61 kb)

## Abbreviations

ACCM: American College of Critical Care Medicine
BLS: Basic life support
CCICCM: Chinese College of Intensive and Critical Care Medicine
CMA: Chinese Medical Association
CMDA: Chinese Medical Doctors’ Association
CoBaTrICE: Competency Based Training in Intensive Care Medicine in Europe collaboration
CSCCM: Chinese Society of Critical Care Medicine
CSICM: Chinese Society of Intensive Care Medicine
ICU: Intensive care unit
MARS: Molecular adsorbent recirculation system
MCQ: multiple-choice question
NG: Nominal group
OSCE: Objective structured clinical examination
SCCM: Society of Critical Care Medicine
