# Supplementary material for: Consensus development of core competencies in intensive and critical care medicine training in China
Source: Crit Care. 2016 Oct 16;20:330. doi: 10.1186/s13054-016-1514-z (PMC5065915; doi:10.1186/s13054-016-1514-z)
Supplement: Additional file 1: — is Table S1. Presenting detailed information for members of the NG, Table S2. Presenting items not identified as core competencies, and Table S3. Presenting a comparison of core competencies generated by the CCCCTG and CoBaTrICE. (DOCX 61 kb) [file 13054_2016_1514_MOESM1_ESM.docx]

**Electronic Data Supplement**

Supplement Table S1. Detailed information of members of nominal group

| Name | Title | Affiliation | Representing Body |
| --- | --- | --- | --- |
| Bin Du | M.D. | Professor and Director, Medical ICU, Peking Union Medical College Hospital, Beijing | CSCCM |
| Xiuming Xi | M.D. | Professor, Department of Critical Care Medicine, Fuxing Hospital, Capital Medical University, Beijing | CCICCM |
| Penglin Ma | M.D. | Professor and Director, Department of Emergency and Critical Care Medicine, People’s Liberation Army 309 Hospital, Beijing | CCICCM |
| Haibo Qiu | M.D. | Professor and Director, Department of Emergency and Critical Care Medicine, Zhongda Hospital, Southeast University, Nanjing, Jiangsu Province | CSICM |
| Kaijiang Yu | M.D. | Professor, Department of Critical Care Medicine, Haerbin Medical University Third Hospital, Haerbin, Heilongjiang Province | CSICM |
| Yimin Li | M.D. | Professor, Department of Pulmonary and Critical Care Medicine, Guangzhou Medical University 1^st^ Affiliated Hospital, Guangzhou, Guangdong Province | CSICM |
| Chuanyun Qian | M.D. | Professor and Director, Department of Emergency and Critical Care Medicine, Kunming Medical University 1^st^ Hospital, Kunming, Yunnan Province | CSCCM |
| Qiang Fang | M.D. | Professor and Director, Department of Critical Care Medicine, Zhejiang University 1^st^ Hospital, Hangzhou, Zhejiang Province | CCICCM |
| Yushan Wang | M.D. | Professor and Director, Department of Critical Care Medicine, Jilin University 1^st^ Hospital, Changchun, Jilin Province | CSCCM |
| Wei He | M.D. | Attending Physician, Department of Critical Care Medicine, Tongren Hospital, Capital Medical University, Beijing |  |
| Chunbo Yang | M.D. | Fellow, Department of Critical Care Medicine, Xinjiang Medical University 1^st^ Hospital, Urumuqi, Xinjiang |  |
| Ruoming Tan | M.D. | Fellow, Surgical ICU, Ruijin Hospital, Shanghai Jiaotong University, Shanghai |  |
| Yan Liao | R.N. | Head Nurse, Department of Critical Care Medicine, West China Hospital, Sichuan University, Chengdu, Sichuan Province |  |
| Jie Li | R.T. | Department of Pulmonary and Critical Care Medicine, Beijing Chaoyang Hospital, Capital Medical University, Beijing |  |

CCICCM, Chinese College of Intensive and Critical Care Medicine; CSCCM, Chinese Society of Critical Care Medicine; CSICM, Chinese Society of Intensive Care Medicine

Supplement Table S2. Items not identified as core competencies

| Themes | Competency statements | Online survey | Agreement^a^ | | | | |
| --- | --- | --- | --- | --- | --- | --- | --- |
|  |  |  | round 1 | round 2 | round 3 | round 4 | round 5 |
| 1. Resuscitation and initial management of acutely ill patient | 1.1 Triages and prioritizes acutely ill patients | 82.4% | 77% | 69% | 62% | 69% | 46% |
|  | 1.2 Assesses and provides initial management of the burn patient | 77.4% | 54% | 23% | 23% | 15% | 15% |
|  | 1.3 Assesses and provides initial management of mass casualty | 81.4% | 85% | 92% | 92% | 46% | 31% |
| 2. Diagnosis, assessment, investigation, monitoring and data interpretation | 2.1 Describes and assesses patient with disease-specific scoring system (e.g. Ranson criteria for severe acute pancreatitis) | 84.4% | 69% | 31% | 38% | 15% | 23% |
|  | 2.2 Performs and interprets microbiological investigation | 68.3% | 62% | 77% | 77% | 77% | 92% |
|  | 2.3 Interprets echocardiography | 64.3% | 92% | 77% | 77% | 23% | 46% |
| 3. Disease management | 3.1 Recognizes and manages acute myocarditis | 89.7% | 85% | 77% | 77% | 54% | 38% |
|  | 3.2 Recognizes and manages cardiac tamponade | 95.2% | 92% | 85% | 92% | 69% | 92% |
|  | 3.3 Assesses and manages acute and chronic life-threatening valvular disorders | 87.4% | 85% | 69% | 62% | 54% | 31% |
|  | 3.4 Assesses and manages near drowning | 90.7% | 85% | 85% | 92% | 77% | 38% |
|  | 3.5 Recognizes and manages hepatorenal syndrome | 91.5% | 85% | 69% | 54% | 38% | 31% |
|  | 3.6 Recognizes and manages cardiorenal syndrome | 91.5% | 85% | 69% | 54% | 38% | 23% |
|  | 3.7 Recognizes and manages critical illness neuromyopathy | 91.5% | 85% | 85% | 92% | 31% | 23% |
|  | 3.8 Assesses and manages neoplasm of central nervous system | 67.3% | 31% | 23% | 15% | 0% | 0% |
|  | 3.9 Assesses and manages swallow dysfunction with central nervous system disorders | 87.4% | 85% | 77% | 92% | 15% | 0% |
|  | 3.10 Assesses and manages pheochromocytoma | 87.7% | 77% | 69% | 62% | 23% | 15% |
|  | 3.11 Assesses and manages thyroid storm and myxedema coma | 96.0% | 92% | 92% | 92% | 46% | 46% |
|  | 3.12 Assesses and manages opportunistic infection in AIDS, recipient of solid organ or bone marrow transplantation | 92.5% | 92% | 77% | 85% | 23% | 15% |
|  | 3.13 Recognizes and manages antiphospholipid syndrome | 70.4% | 31% | 23% | 15% | 0% | 0% |
|  | 3.14 Manages antithrombotic treatment in malignancy | 71.9% | 31% | 15% | 23% | 23% | 15% |
|  | 3.15 Recognizes and manages mesenteric ischemia/infarction | 93.0% | 92% | 85% | 85% | 38% | 38% |
|  | 3.16 Recognizes and manages gastrointestinal perforation | 94.0% | 92% | 92% | 92% | 62% | 69% |
| 4. Therapeutic interventions/organ system support in single or multiple organ failure | 4.1 Describes principles and practice of immunonutrition | 81.4% | 85% | 77% | 77% | 15% | 15% |
|  | 4.2 Monitors toxicity for vasoactive medication | 96.7% | 92% | 92% | 92% | 62% | 69% |
|  | 4.3 Provides plasmapheresis for acute illness (including neurologic and hematologic diseases) | 93.0% | 85% | 69% | 69% | 15% | 23% |
|  | 4.4 Performs bedside ultrasound for vascular localization | 45.2% | 38% | 38% | 46% | 38% | 31% |
| 5. Practical procedures | 5.1 Performs focused abdominal sonography in trauma (FAST) | 52.8% | 62% | 69% | 69% | 54% | 38% |
|  | 5.2 Performs lung ultrasound | 40.5% | 54% | 62% | 54% | 38% | 31% |
|  | 5.3 Performs transthoracic echocardiography (TTE) | 39.4% | 46% | 54% | 38% | 23% | 8% |
|  | 5.4 Performs transesophageal echocardiography (TEE) | 22.6% | 31% | 15% | 31% | 8% | 0% |
|  | 5.5 Performs percutaneous tracheostomy | 82.9% | 85% | 85% | 92% | 54% | 85% |
|  | 5.6 Performs fibrotic bronchoscopy | 88.4% | 85% | 77% | 69% | 54% | 77% |
|  | 5.7 Performs bronchoalveolar lavage | 75.6% | 62% | 38% | 46% | 31% | 62% |
|  | 5.8 Manages advanced respiratory support including high-frequency oscillation, split lung ventilation, and tracheal gas insufflation | 79.4% | 31% | 38% | 31% | 8% | 23% |
|  | 5.9 Interprets capnography | 87.7% | 62% | 46% | 54% | 31% | 23% |
|  | 5.10 Manages respiratory mechanics monitoring | 91.0% | 92% | 85% | 92% | 54% | 69% |
|  | 5.11 Performs peripheral vein cannulation | 78.1% | 77% | 77% | 54% | 38% | 31% |
|  | 5.12 Performs pericardiocentesis in acute tamponade | 74.1% | 54% | 46% | 46% | 38% | 38% |
|  | 5.13 Interprets dynamic electrocardiogram | 66.8% | 54% | 62% | 54% | 31% | 38% |
|  | 5.14 Performs pulmonary artery catheterization | 79.4% | 85% | 77% | 69% | 31% | 62% |
|  | 5.15 Performs arterial pulse contour analysis | 90.7% | 92% | 69% | 69% | 38% | 69% |
|  | 5.16 Performs gastric tonometry | 64.8% | 15% | 31% | 31% | 15% | 0% |
|  | 5.17 Performs transcutaneous or transvenous pacing | 59.8% | 31% | 38% | 31% | 15% | 8% |
|  | 5.18 Performs intra-aortic balloon pump (IABP) | 56.8% | 38% | 38% | 38% | 23% | 15% |
|  | 5.19 Performs extracorporeal membrane oxygenation (ECMO) | 61.3% | 46% | 38% | 46% | 8% | 15% |
|  | 5.20 Administrates analgesia via an epidural catheter | 39.9% | 23% | 23% | 15% | 15% | 0% |
|  | 5.21 Manages intracranial pressure monitoring | 59.5% | 54% | 54% | 62% | 31% | 15% |
|  | 5.22 Performs Sengstaken-Blakemore tube placement | 73.1% | 62% | 62% | 54% | 38% | 23% |
|  | 5.23 Manages intermittent hemodialysis (IHD) | 53.8% | 62% | 62% | 54% | 31% | 15% |
|  | 5.24 Manages peritoneal dialysis (PD) | 33.7% | 23% | 23% | 8% | 31% | 8% |
|  | 5.25 Manages sustained low-efficiency daily diafiltration (SLEDD-f) | 44.0% | 38% | 31% | 31% | 8% | 8% |
|  | 5.26 Manages blood perfusion | 76.1% | 69% | 62% | 46% | 38% | 31% |
|  | 5.27 Manages plasmapheresis | 69.6% | 62% | 46% | 38% | 15% | 15% |
|  | 5.28 Manages molecular adsorbent recirculating system (MARS) | NA | 23% | 23% | 0% |  |  |
| 6. perioperative care | 6.1 Assesses and manages perioperative patients with liver dysfunction | 89.90% | 77% | 77% | 62% | 23% | 23% |
|  | 6.2 Manages post-operative assessment and care of cardiosurgery patient | 78.4% | 38% | 46% | 46% | 8% | 0% |
|  | 6.3 Manages post-operative assessment and care of neurosurgery patient | 89.2% | 46% | 54% | 62% | 23% | 15% |
|  | 6.4 Manages post-operative assessment and care of solid organ-transplant patient | 66.3% | 23% | 15% | 23% | 8% | 8% |
| 7. comfort, recovery and end-of-life care | 7.1 Manages patients under Do Not Resuscitate (DNR) condition | 87.7% | 92% | 85% | 85% | 62% | 62% |
|  | 7.2 Manages the process of withhold or withdraw life-sustaining therapy | 88.7% | 85% | 69% | 77% | 46% | 38% |
|  | 7.3 Manages palliative care of the critically ill patient | 90.2% | 85% | 62% | 69% | 31% | 15% |
|  | 7.4 Manages physiological supportive therapy for organ donors | 71.4% | 38% | 31% | 31% | 31% | 8% |
|  | 7.5 Performs brainstem death testing | 95.5% | 92% | 85% | 92% | 85% | 77% |
| 8. Transport | 8.1 Performs inter-hospital transport | 90.2% | 85% | 77% | 69% | 69% | 62% |
| 9. Patient safety and system management | 9.1 Leads a daily multidisciplinary ward round | 77.9% | 54% | 46% | 38% | 23% | 23% |
| 10. Professionalism | 10.1 Describes and explains principles of informed consent | 98.0% | 92% | 92% | 92% | 92% | 85% |
|  | 10.2 Supports clinical staff outside the ICU to enable the delivery of effective care | 93.2% | 77% | 69% | 69% | 62% | 54% |
|  | 10.3 Participates in multidisciplinary teaching | 92.7% | 92% | 92% | 92% | 62% | 54% |
|  | 10.4 Participates in clinical research | 91.2% | 92% | 85% | 85% | 38% | 46% |
| 11. Certification | 11.1 Advanced Trauma Life Support (ATLS) | 91.7% | 92% | 92% | 92% | 77% | 69% |

FAST: Focused Abdominal Sonography; TTE: Transthoracic echocardiography; TEE: Transesophageal echocardiography; IABP: Intra-Aortic Balloon Pump; ECMO: Extracorporeal Membrane Oxygenation; IHD: Intermittent Hemodialysis; PD: Peritoneal Dialysis; SLEDD-f: Sustained Low-Efficiency Daily Diafiltraiton; MARS: Molecular Adsorbent Recirculating System; DNR: Do Not Resuscitate; ATLS: Advanced Trauma Life Support

^a^Percentage of respondents rating agree or strongly agree

Supplement Table S3. Comparison of core competencies generated by CCCCTG and CoBaTrICE

| Theme | Competency statement^a^ | CCCCTG^b^ | CoBaTrICE^b^ |
| --- | --- | --- | --- |
| 1. Resuscitation and initial management of acutely ill patient | Assesses and provides initial management of the trauma patient | √ | √ |
|  | Manages cardiopulmonary resuscitation | √ | √ |
|  | Manages post-resuscitation brain protection | √ |  |
|  | Provides advanced life support for post-resuscitation patient | √ | √ |
|  | Triages and prioritises patients appropriately, including timely admission to ICU |  | √ |
|  | Assesses and provides initial management of the patient with burns |  | √ |
|  | Describes the management of mass casualties |  | √ |
| 2. Diagnosis, assessment, investigation, monitoring and data interpretation | Obtains medical history and performs accurate physical examination | √ | √ |
|  | Orders timely and appropriate laboratory investigations | √ | √ |
|  | Orders timely and appropriate image investigations | √ | √ |
|  | Integrates clinical findings with laboratory investigations to form a differential diagnosis | √ | √ |
|  | Describes and assesses patient with severity-of-illness score: APACHE, SAPS | √ | √ |
|  | Describes and assesses patient with multi-organ dysfunction score: SOFA, MODS | √ |  |
|  | Describes monitoring and interpretation of respiratory mechanics | √ |  |
|  | Interprets arterial blood gas analysis | √ | √ |
|  | Performs electrocardiography and interprets the results | √ | √ |
|  | Interprets chest radiographs | √ | √ |
|  | Interprets CT image | √ |  |
|  | Describes indications for echocardiography (transthoracic, transoesophageal) |  | √ |
|  | Obtains appropriate microbiological samples and interprets results |  | √ |
|  | Liaises with radiologists to organise and interpret clinical imaging |  | √ |
|  | Monitors and responds to trends in physiological variables |  | √ |
| 3. Disease management | Describes implications of chronic and comorbid disease in the acutely ill patient | √ | √ |
|  | Recognizes and manages different types of shock | √ | √ |
|  | Assesses and manages life threatening arrhythmia | √ |  |
|  | Recognized and manages left ventricular failure and/or acute pulmonary edema | √ |  |
|  | Recognizes and manages right heart failure | √ |  |
|  | Assesses and manages myocardial infarction and acute coronary syndrome | √ |  |
|  | Recognizes and manages rupture of aneurysm (bleeding and cardiac tampondae) | √ |  |
|  | Recognizes and manages hypertension crisis | √ |  |
|  | Describes physiological changes of cardiovascular system under acute condition | √ |  |
|  | Assesses and manages acute and chronic respiratory failure | √ |  |
|  | Assesses and manages acute exacerbation of chronic obstructive pulmonary disease | √ |  |
|  | Assesses and manages status asthmaticus | √ |  |
|  | Assesses and manages smoke inhalation, airway burns | √ |  |
|  | Assesses and manages upper airway obstruction (due to infection or foreign body) | √ |  |
|  | Recognizes (diagnosis and grading) and manages acute respiratory distress syndrome (ARDS) | √ | √ |
|  | Manages life threatening hemoptysis | √ |  |
|  | Describes effects of positioning on respiratory physiology | √ |  |
|  | Recognizes (diagnosis and grading) and manages acute kidney injury | √ | √ |
|  | Manages critically ill patients with chronic renal failure | √ |  |
|  | Manages patients with coma | √ | √ |
|  | Assesses and manages patients with drug overdose and intoxication | √ | √ |
|  | Assesses and manages cerebral vascular accident | √ |  |
|  | Manages status epilepticus | √ |  |
|  | Recognizes and manages intracranial infection | √ |  |
|  | Assesses and manages patient with increased intracranial pressure | √ |  |
|  | Assesses and manages spine injury | √ |  |
|  | Recognizes and manages adrenal crisis | √ |  |
|  | Recognizes and manages diabetes insipidus | √ |  |
|  | Recognizes and manages diabetic ketoacidosis | √ |  |
|  | Recognizes and manages sepsis, severe sepsis and septic shock | √ | √ |
|  | Assesses and manages multi-organ dysfunction syndrome | √ |  |
|  | Assesses and manages severe community acquired infection (e.g. severe community acquired pneumonia) | √ |  |
|  | Recognizes and manages nosocomial infection | √ |  |
|  | Assesses and manages fever in critically ill patient | √ |  |
|  | Describes antimicrobial resistance | √ |  |
|  | Recognizes intra-abdominal infection and gastrointestinal leakage | √ |  |
|  | Manages coagulopathy | √ |  |
|  | Manages hemolytic disorders | √ |  |
|  | Assesses and manages thromboembolic disease (including pulmonary embolism) | √ |  |
|  | Manages disseminated intravascular coagulation | √ |  |
|  | Manages traumatic coagulopathy | √ |  |
|  | Assesses and manages gastrointestinal bleeding | √ | √ |
|  | Assesses and manages patient with liver failure | √ | √ |
|  | Assesses and manages pancreatitis | √ |  |
|  | Assesses and manages abdominal compartment syndrome | √ |  |
|  | Assesses and manages acute illness in pregnancy | √ | √ |
| 4. Therapeutic interventions/organ system support in single or multiple organ failure | Assesses and manages fluid and electrolyte disorders | √ | √ |
|  | Assesses and manages acid-base disorders | √ | √ |
|  | Describes and provides parenteral nutrition support | √ | √ |
|  | Describes and provides enteral nutrition support | √ | √ |
|  | Provides nutrition support for patient with severe acute pancreatitis | √ |  |
|  | Provides nutrition support for patient with renal failure | √ |  |
|  | Provides nutrition support for patient with liver failure | √ |  |
|  | Provides nutrition support for patient with sepsis and septic shock | √ |  |
|  | Provides nutrition support for post gastrointestinal surgery patient | √ |  |
|  | Assesses and manages pain in critically ill patients | √ | √ |
|  | Describes principle and assessment of sedation | √ |  |
|  | Provides assessment, prevention and treatment of delirium | √ | √ |
|  | Describes indication and choice of neuromuscular blockade | √ | √ |
|  | Manages fluid therapy | √ | √ |
|  | Manages vasoactive/inotropic medication therapy | √ | √ |
|  | Describes principles of drug dose adjustment in renal failure | √ |  |
|  | Describes principles of continuous renal replacement therapy | √ | √ |
|  | Explains and appraises management of severe sepsis and septic shock | √ |  |
|  | Describes principle of antimicrobial agent selection and dosing in critically ill patients | √ | √ |
|  | Describes principle of anticoagulation; anti-fibrnolytic therapy | √ |  |
|  | Describes principle of blood component transfusion | √ | √ |
|  | Describes stress ulcer prophylaxis | √ |  |
| 5. Practical procedures | Performs bedside ultrasound to localize pleural effusion and ascites | √ |  |
|  | Maintains an open airway in the non-intubated patient | √ | √ |
|  | Performs bag-mask ventilation | √ | √ |
|  | Performs tracheal intubation | √ | √ |
|  | Performs tracheal aspiration | √ | √ |
|  | Manages pneumothorax | √ |  |
|  | Administers oxygen therapy | √ | √ |
|  | Manages non-invasive and invasive mechanical ventilation: indication, rational, complication and weaning | √ | √ |
|  | Explains and performs recruitment maneuver: principle and practice | √ |  |
|  | Performs arterial puncture and cannulation | √ | √ |
|  | Performs central venous catheters insertion | √ | √ |
|  | Performs and interprets cardiac output and hemodynamic monitor | √ | √ |
|  | Performs cardioversion and defibrillation | √ | √ |
|  | Performs lumber puncture | √ | √ |
|  | Performs nasogastric tube placement | √ | √ |
|  | Performs abdominal paracentesis | √ | √ |
|  | Performs and interprets intra-abdominal pressure monitor | √ |  |
|  | Manages continuous renal replacement therapy | √ |  |
|  | Performs urinary catheterization | √ |  |
|  | Performs fiberoptic laryngoscopy under supervision |  | √ |
|  | Performs difficult and failed airway management according to local protocols |  | √ |
|  | Performs fiberoptic bronchoscopy and bronchoalveolar lavage in the intubated patient under supervision |  | √ |
|  | Performs percutaneous tracheostomy under supervision |  | √ |
|  | Performs thoracocentesis via a chest drain |  | √ |
|  | Performs peripheral venous catheterization |  | √ |
|  | Describes a method for surgical isolation of vein/artery |  | √ |
|  | Performs cardiac pacing (Transvenous or transthoracic) |  | √ |
|  | Describes how to perform pericardocentesis |  | √ |
|  | Manages the administration of analgesia via an epidural catheter |  | √ |
|  | Describes Sengstaken tube (or equivalet) placement |  | √ |
|  | Describes indications for, and safe conduct of gastroscopy |  | √ |
|  | Performs urinary catheterization |  | √ |
| 6. perioperative care | Performs pre-operative cardiopulmonary evaluation of high-risk patient | √ | √ |
|  | Manages post-operative assessment and care of high-risk surgical patient | √ | √ |
|  | Manages the pre- and post-operative care of the trauma patients | √ | √ |
|  | Manages the care of the patient following cardiac surgery under supervision |  | √ |
|  | Manages the care of the patient following craniotomy under supervision |  | √ |
|  | Manages the care of the patient following solid organ transplantation under supervision |  | √ |
| 7. comfort, recovery and end-of-life care | Describes and applies practice to minimizes the physical and psychosocial consequences of critical illness for patients and families | √ | √ |
|  | Manages the safe and timely discharge of patients from the ICU | √ | √ |
|  | Communicates the continuing care requirements of patients at ICU discharge to health care professionals, patients and relatives | √ | √ |
|  | Manages the process of witholding orwithdrawing treatment with the multidisciplinary team |  | √ |
|  | Manages palliative care of the critically ill patient |  | √ |
|  | Performs brainstem death testing |  | √ |
|  | Manages the paysiological support of the organ donor |  | √ |
| 8. Transport | Assesses patient  before transport | √ |  |
|  | Prepares equipment for transport | √ |  |
|  | Performs intra-hospital transport | √ | √ |
| 9. Patient safety and system management | Complies with infection control measures | √ | √ |
|  | Identifies environmental hazards and promotes safety for patients and staff | √ | √ |
|  | Organizes a case conference | √ | √ |
|  | Critically appraises and applies guidelines, protocols and card bundles | √ | √ |
|  | Leads daily multidisciplinary word round |  | √ |
|  | Identifies and minimizes risk of critical incidents and adverse events, including complications of critical illness |  | √ |
|  | Demonstrates an understanding of the managerial and administrative responsibilities of the ICM specialist |  | √ |
|  | Describes commonly used scoring systems for assessment of case mix and workload |  | √ |
| 10. Professionalism | Formulates clinical decisions with respect for ethical and legal principles | √ | √ |
|  | Involves patients (or their surrogates if applicable) in decisions about  care and treatment (including informed consent and end-of-life care) | √ | √ |
|  | Demonstrates respect of cultural and religious beliefs and an awareness of their impact on decision making | √ | √ |
|  | Promotes effective team working | √ | √ |
|  | Communicates effectively with patients and relatives | √ | √ |
|  | Communicates effectively with members of the health care team | √ | √ |
|  | Maintains accurate medical records and documentation | √ | √ |
|  | Respects privacy, dignity, confidentiality and legal constraints on the use of patient data | √ | √ |
|  | Takes responsibility for safe patient care | √ | √ |
|  | Ensures continuity of care through effective hand-over of clinical information | √ | √ |
|  | Seeks learning opportunities and integrates new knowledge into  clinical practice | √ | √ |
|  | Describes and  explains the managerial and administrative responsibilities of the ICM specialist | √ |  |
|  | Supports clinical staff outside the ICU to enable the delivery of effective care |  | √ |
|  | Appropriately supervises, and delegates to other, the delivery of patient care |  | √ |
|  | Participates in multidisciplinary teaching |  | √ |
|  | Participates in research or audit under supervision |  | √ |
| 11. Certification | Basic Life Support (BLS) | √ |  |
|  | Advanced Cardiac Life Support (ACLS) | √ |  |

^a^Statements and domains are presented on the basis of competencies generated by CCCCTG, statements existed only in CoBaTrICE' list are listed in domains most close to their original assignment.

^b^Check mark (√) was given when the key words of the statement existed in corresponding list ingoring the level of expertise required.
